# Impulsivity, Cognitive Distortions, and Problem Gambling Among Spanish Adults Who Gamble: Exploring the Moderating Role of Gambling Self-Efficacy

**DOI:** 10.3390/ejihpe16050065

**Published:** 2026-05-07

**Authors:** Ainhoa Coloma-Carmona, Cristina J. Valdivia Blanco, José Luis Carballo, Antonia Pelegrín-Muñoz

**Affiliations:** 1 Center for Applied Psychology, Miguel Hernández University of Elche, 03202 Elche, Spain; cristina.valdivia01@goumh.umh.es (C.J.V.B.); jcarballo@umh.es (J.L.C.); 2 Brief Intervention and Addictions Group (IBREA), Miguel Hernández University of Elche, 03202 Elche, Spain; apelegrin@umh.es; 3 Alicante Institute for Health and Biomedical Research (ISABIAL), 03010 Alicante, Spain

**Keywords:** cognitive distortions, gambling-like activities, gambling self-efficacy, general population, impulsivity, moderation, problem gambling

## Abstract

Gambling self-efficacy is a key construct in understanding gambling behavior and behavior change. This study examined (i) differences between individuals with low and high gambling self-efficacy and (ii) tested whether gambling self-efficacy moderated the associations between impulsivity, gambling-related cognitive distortions, and problem gambling severity. Data were drawn from a cross-sectional web-based survey of 1429 Spanish adults aged 18–64. Analyses focused on the 921 who had engaged in gambling activities during the previous 12 months (mean age = 40 years, SD = 12.1, 52.2% men). Measures included sociodemographic characteristics, gambling self-efficacy (BSCQ-G), problem gambling severity (PGSI), impulsivity (UPPS-P), gambling-related cognitive distortions (Labrador’s cognitive distortion scale), and gambling involvement indicators. Participants were classified into low (BSCQ-G < 80%; 51.5%; *n* = 474) and high (BSCQ-G ≥ 80%; 48.5%; *n* = 447) gambling self-efficacy groups. Individuals with low self-efficacy showed broader and more intensive gambling involvement, including greater participation in gambling-like activities, and higher scores on PGSI, impulsivity, and gambling-related cognitive distortions. In moderation models adjusted for gambling involvement indicators, the association between impulsivity and PGSI was weaker at higher levels of gambling self-efficacy, although the incremental variance explained was very small (ΔR^2^ = 0.0024). The interaction between gambling self-efficacy and cognitive distortions was not statistically supported. These findings suggest that gambling self-efficacy is associated with gambling risk profiles among adults who gamble and may play a modest buffering role in the association between impulsivity and gambling severity.

## 1. Introduction

Gambling behavior and problem gambling have emerged as significant public health concerns (Wardle et al., 2024). Problem gambling is associated with a wide range of harmful consequences, including financial difficulties, strained social relationships, intimate partner violence, job loss, and over-indebtedness (Browne et al., 2018; Håkansson & Widinghoff, 2020; Price et al., 2021; Raybould et al., 2021; Wall et al., 2025). In severe cases, it has been linked to suicidal behavior (Armoon et al., 2023; Kesaite et al., 2024; Koompah et al., 2024; Kristensen et al., 2024; Marionneau et al., 2023; O’Sullivan, 2023; Tulloch et al., 2024; Wardle et al., 2023). Understanding the factors that contribute to greater or lesser involvement in gambling activities, as well as to the development of problem gambling, is therefore essential for designing effective prevention and intervention strategies (Allami et al., 2021; León-Jariego et al., 2020).

While self-efficacy is broadly defined as an individual’s belief in their capacity to successfully perform specific behaviors (Bandura, 1977), its application in addiction research is commonly operationalized in domain-specific and situational terms. In this context, and particularly within Marlatt’s relapse prevention model, self-efficacy refers to an individual’s confidence in resisting urges and maintaining behavior change in high-risk situations (Marlatt & Gordon, 1985). Accordingly, gambling-specific self-efficacy is often defined as the confidence in one’s ability to resist the urge to gamble across various specific high-risk situations (Casey et al., 2008).

Gambling self-efficacy has been demonstrated to play a significant role in problem gambling (Challet-Bouju et al., 2025; Hawker et al., 2021; Stark, 2014; Wu et al., 2016) and in predicting gambling behavior (Hawker et al., 2021; Oei & Raylu, 2015). Evidence suggests that gambling self-efficacy facilitates the distinction between non-problem and problem gamblers (Casey et al., 2008; Winfree et al., 2014), and plays a protective role by reducing the probability of gambling urges and by enhancing motivation to sustain gambling avoidance in high-risk situations (Challet-Bouju et al., 2025; Dowling et al., 2022, 2021c; Free et al., 2024; Hawker et al., 2021; He & Tong, 2024; Valencia & Peters, 2025; Barbaranelli et al., 2017; Mishra et al., 2019; Quinn et al., 2019; Ridder & Deighton, 2022; Wu et al., 2016). Gambling self-efficacy is also commonly included as a fundamental mechanism of change in evaluations of gambling interventions (Dowling et al., 2021b; Magill et al., 2025; Mansueto et al., 2024; Valencia & Peters, 2025) and has been associated with treatment success, relapse prevention, and risk management (Dowling et al., 2021b; Ridder & Deighton, 2022).

Consistent with this evidence, research has identified lower levels of gambling self-efficacy as a significant factor contributing to problem gambling, linked to higher gambling expenditures and longer gambling sessions (Hawker et al., 2021). Insufficient confidence in regulating gambling behavior is associated with greater severity and frequency of problem gambling (León-Jariego et al., 2020; Quinn et al., 2019; Winfree et al., 2014) and higher relapse rates and poorer treatment outcomes (Hawker et al., 2021). Furthermore, the inability to resist the urge to gamble is linked to a greater perceived availability of gambling, which exacerbates gambling-related problem behaviors (Parrado-González et al., 2022). In contrast, individuals with high gambling self-efficacy tend to gamble less frequently (Hodgins et al., 2004; León-Jariego et al., 2020), experience fewer gambling-related problems (St-Pierre et al., 2015), demonstrate greater behavioral control (Iliceto et al., 2021), and engage in more responsible gambling practices (He & Tong, 2024).

Beyond its direct association with gambling severity, gambling self-efficacy has been suggested to attenuate the impact of both environmental and psychological risk factors. Higher levels of gambling self-efficacy are associated with a reduced strength of the relationship between perceived advertising and problem gambling (Quinn et al., 2019), between high-risk situations and gambling involvement, and between momentary craving and gambling expenditure or episode occurrence (Hawker et al., 2021). Evidence also indicates that lower gambling self-efficacy may intensify the association between coping-motivated gambling and spending (Hawker et al., 2021), pointing to increased vulnerability when confidence to resist gambling is diminished. Converging evidence further conceptualizes gambling self-efficacy as shaping how risk states translate into behavior, highlighting its potential role in modifying the strength of associations between psychological vulnerabilities and gambling outcomes (Challet-Bouju et al., 2025; Dowling et al., 2021b; Free et al., 2024; He & Tong, 2024). Moreover, within the framework of the Theory of Planned Behavior, gambling self-efficacy has also been linked to lower gambling frequency indirectly through its influence on gambling intentions (Martin et al., 2010), suggesting a regulatory role operating at both situational, behavioral, and cognitive levels.

Although self-efficacy has been explored in gambling research and the broader field of addictive behaviors (Bandura, 1977; Carballo et al., 2025; DiClemente et al., 1995; He & Tong, 2024; Mishra et al., 2019; Parrado-González et al., 2022; Quinn et al., 2019; Ridder & Deighton, 2022; Sancho-Domingo et al., 2025; Torrejón-Guirado et al., 2025), research on its role among adults who gamble in the general population remains limited, as most studies focus on clinical samples and conceptualize gambling self-efficacy as a treatment-related variable (Challet-Bouju et al., 2025; Free et al., 2024; Hawker et al., 2021). Further investigation is required to determine whether this construct is also associated with broader gambling involvement outside treatment settings, particularly with participation in new gambling formats embedded in digital environments.

In this regard, gambling-like activities merit particular consideration because digital technologies, in addition to making online gambling widely accessible, have expanded access to products that incorporate gambling-related mechanics outside traditional gambling settings (Allami et al., 2021; Andrade & Newall, 2023; Hing et al., 2024; Macey et al., 2024; Macey & Hamari, 2024). Recent studies have indicated that participation in such activities is associated with greater gambling engagement and gambling-related problems (Chóliz et al., 2021; Coloma-Carmona et al., 2024b, 2026b; Hing et al., 2023b; Lopez-Gonzalez & Petrotta, 2024; Wardle & Tipping, 2023). Among the most widely studied gambling-like or gambling-adjacent formats are loot boxes (Brooks & Clark, 2019; Hing et al., 2022a; Yokomitsu et al., 2021; Zendle, 2020), esports betting and virtual item betting, including skin gambling (Delfabbro & King, 2023; Hing et al., 2023a; King & Delfabbro, 2020), and digital assets such as cryptocurrencies, NFTs, or fan tokens (Coloma-Carmona et al., 2024b; Delfabbro et al., 2021; Delfabbro & King, 2023; Grubbs & Kraus, 2023; Johnson et al., 2023; Lopez-Gonzalez & Petrotta, 2024; Louderback et al., 2024). These developments are especially visible in digital environments such as video games and streaming platforms, where gambling-like mechanics and gambling-analogous products increasingly overlap with entertainment and monetization systems (Coloma-Carmona et al., 2024a, 2024b; Gibson et al., 2023; Griffiths & Nuyens, 2017; Steinmetz et al., 2022; Turner & Shi, 2023).

Evidence suggests that certain profiles, particularly those marked by heightened impulsivity and gambling-related cognitive distortions, are more prone to engage in new gambling formats (Barrault & Varescon, 2023; Brooks & Clark, 2019; Coloma-Carmona et al., 2024b; Leslie et al., 2025; Wardle & Zendle, 2021). However, the role of gambling self-efficacy in engagement with these gambling-like activities remains underexplored. This gap is relevant because impulsivity and gambling-related cognitive distortions have been consistently identified as central variables in the development and maintenance of gambling disorder (Blaszczynski & Nower, 2002; Browne et al., 2019; Dowling et al., 2021a; Mishra et al., 2019; Nower et al., 2022). Cognitive distortions have been described as an integral component of the development, maintenance, and treatment of gambling disorder, with meta-analytic evidence showing robust differences between individuals with and without gambling problems (Goodie & Fortune, 2013). Impulsivity is likewise considered a central, multifaceted construct that represents a relevant behavioral dysregulation pathway through which difficulties in inhibitory control, decision-making, and impulse regulation may contribute to gambling problems (Ioannidis et al., 2019). Both constructs are associated with problem gambling and increased gambling involvement (Choi & Kim, 2021; Devos et al., 2020; Hing & Russell, 2020; Labrador et al., 2020; Leonard et al., 2021; Mestre-Bach et al., 2020, 2021; Richard & King, 2023; Tan & Tam, 2024; Williams et al., 2023) and are involved in the onset and persistence of problem gambling (Devos et al., 2020; Iliceto et al., 2021).

Against this background, and given the role of self-efficacy in the maintenance and modification of addictive behaviors, as well as in treatment outcomes (Bandura, 1977; DiClemente et al., 1995; Hawker et al., 2021; Lai et al., 2015; Oei & Goh, 2015), further research is needed to clarify its relevance outside clinical or treatment-seeking contexts. Moreover, there is scarce evidence about whether gambling self-efficacy is associated with gambling involvement, including the engagement in gambling-like activities, within the general population, or whether it modifies the association between established psychological vulnerability factors and problem gambling. The present study aimed to (i) describe differences between individuals with low versus high gambling self-efficacy in sociodemographic characteristics, gambling involvement (including emerging formats), problem gambling severity, impulsivity, and gambling-related cognitive distortions; and (ii) examine whether gambling self-efficacy moderates the associations of impulsivity and gambling-related cognitive distortions with problem gambling severity, adjusting for indicators of gambling involvement that differ across self-efficacy profiles. Based on previous evidence, we expected individuals with low gambling self-efficacy to show greater gambling involvement, greater engagement in gambling-like activities, higher problem gambling severity, higher impulsivity, and stronger gambling-related cognitive distortions than those with high gambling self-efficacy. We further expected gambling self-efficacy to moderate the associations between both psychological risk factors and problem gambling severity, such that these associations would be weaker at higher levels of gambling self-efficacy.

## 2. Materials and Methods

### 2.1. Participants

The study sample comprised 921 Spanish adults aged 18–64 years who reported engaging in at least one gambling activity (e.g., lotteries, sports betting, casino, card games) during the previous 12 months. These participants represented 64% of the valid initial survey sample.

As shown in Table 1, the mean age of participants was 40 years (SD = 12.1). The sample exhibited a balanced gender distribution, with 52.2% male (*n* = 481) and 47.8% (*n* = 440) female participants. A significant proportion of the participants reported having attained secondary (44.1%; *n* = 406) and higher education (50.6%; *n* = 466), and most were employed (69.3%; *n* = 638). Regarding monthly income, among the participants who provided this information, the majority reported earning between 1000 and 1,999 euros (55.9%, *n* = 391). Additionally, 20.9% (*n* = 146) earned less than 1000 euros, and 23.3% (*n* = 163) reported an income of 2000 euros or more. Finally, 24% (*n* = 221) of participants did not provide information about their income.

Regarding gambling involvement (Table 2), 42.7% (*n* = 393) of participants gambled less than once a month, 34.7% (*n* = 320) gambled monthly, 20.3% (*n* = 187) reported weekly gambling, and 2.3% (*n* = 21) gambled daily. Participants engaged in an average of 1.5 (SD = 1.1) gambling activities over the past 12 months. Regarding new formats, 8% (*n* = 74) of the sample reported engaging in gambling-like activities, with an average of 0.2 (SD = 0.6) activities specifically related to video games or streaming platforms. Moreover, the prevalence of problem gambling (PGSI ≥ 5) in the total sample was 6.7% (*n* = 62), with a mean PGSI score of 1.1 (SD = 3.1).

### 2.2. Design and Procedure

This study adopted a cross-sectional, descriptive-correlational design, based on a single measurement obtained from participants. Data were collected through a web-based questionnaire administered between 24 March and 22 April 2022.

Participants were recruited through a random selection process from an online panel managed by a Spanish independent agency specialized in exploratory and ad hoc qualitative and quantitative research. Sampling quotas were applied according to age, sex, geographic area, and habitat size in order to approximate the distribution of the Spanish adult population. Data-quality procedures were applied before deriving the final valid sample. Responses were excluded if they were incomplete, completed too rapidly, exceeded quota limits, showed inconsistencies, or presented automated/infrequent response patterns, identified using the dichotomous format of the Oviedo Infrequency Scale (Fonseca-Pedrero et al., 2019). Participants with two or more incorrect responses on this scale were considered to have provided random or inattentive responses. After applying these procedures, the valid initial sample comprised 1429 Spanish adults aged 18–64 years.

Participation was voluntary, and all respondents provided informed consent prior to completing the survey. The study was conducted in accordance with the Declaration of Helsinki and was approved by the Committee of Research and Ethics of the University Miguel Hernández de Elche (reference: DPS.ACC.01.21).

### 2.3. Measures

#### 2.3.1. Sociodemographic Variables

Data pertaining to sex, age, educational level, employment status, and monthly income were collected.

#### 2.3.2. Gambling Self-Efficacy

The Brief Situational Confidence Questionnaire for Gambling (BSCQ-G; Coloma-Carmona et al., 2026a) was used to assess perceived self-efficacy in resisting the urge to gamble in high-risk situations. This instrument comprises ten items, with respondents rating their confidence on a scale ranging from 0% to 100% (0 = “not at all confident”, 100 = “completely confident”) to assess confidence in avoiding gambling in ten high-risk gambling situations. A cutoff score of ≥ 80% was used to categorize participants into low and high self-efficacy groups. This threshold has demonstrated a sensitivity of 93.5% and specificity of 51.6% for identifying individuals with non-problem gambling. In this sample, the BSCQ-G demonstrated excellent reliability (α and ω = 0.98), and a unidimensional structure, as confirmed through exploratory and confirmatory factor analyses (Coloma-Carmona et al., 2026a).

#### 2.3.3. Engagement in Gambling Activities

Participants reported their involvement in eight specific gambling practices during the past 12 months (yes/no), covering activities assessed in Spanish national surveys on gambling behaviors (Spanish Observatory on Drugs and Addictions, 2023). For each activity, participants indicated whether it was conducted online, in a land-based setting, or both: (1) lotteries, coupons, pools, and scratch cards, (2) sports betting, (3) horse race betting, (4) slot machines and gaming machines, (5) card games, (6) bingo, (7) casino games or gaming rooms, and (8) contests with monetary bets. From these data, three variables were derived. First, the total number of gambling activities was calculated. Second, gambling frequency was determined by the highest frequency reported for any single activity, categorized as: less than once a month, monthly, weekly, and daily. Third, to identify the preferred form of gambling, the total counts of land-based and online activities were compared to determine the most prevalent format for each participant.

#### 2.3.4. Engagement in Gambling-like Activities

Participants reported whether they had participated (yes/no) during the preceding twelve months in various activities within video games and streaming platforms that incorporate gambling elements (Zendle, 2020). These included: (1) betting on eSports, (2) skin gambling on video games, (3) purchasing loot boxes, and (4) betting on live streaming video games.

#### 2.3.5. Problem Gambling

Severity of gambling behaviors was assessed using the Spanish adaptation of the Problem Gambling Severity Index (PGSI; Ferris, 2001; Lopez-Gonzalez et al., 2018). The PGSI comprises nine items, each rated on a four-point Likert scale, with higher scores indicating greater gambling problem severity. A score of 5 or higher was used to identify individuals with problem gambling (Williams & Volberg, 2014). This instrument has demonstrated high internal reliability (ordinal α = 0.97) and strong criterion validity with DSM-IV criteria (*r* = 0.77). In the present study, the internal consistency was ω = 0.88.

#### 2.3.6. Problem Gambling Risk Factors

The assessment included impulsivity and gambling-related cognitive distortions, as these constructs are well-established risk factors for problem gambling (Browne et al., 2019; Dowling et al., 2021a; Mishra et al., 2019; Nower et al., 2022). Impulsivity was assessed using the Spanish adaptation of the UPPS-P Impulsive Behavior Scale (Billieux et al., 2012). This 20-item instrument, rated on a four-point Likert scale (Cándido et al., 2012), evaluates five impulsivity domains: negative urgency, lack of premeditation, lack of perseverance, sensation seeking, and positive urgency. In addition, a global impulsivity score was also calculated, with higher scores reflecting greater impulsivity. The scale has demonstrated satisfactory psychometric properties, with reliability coefficients ranging from α = 0.61–0.81 (Cándido et al., 2012). In the present study, the internal consistency was ω = 0.85. Gambling-related cognitive distortions were measured using the Labrador et al. (2020) scale. This 9-item instrument assesses six common cognitive biases: illusion of control, illusory correlation, luck as responsible for outcomes, self-correcting randomness, biased evaluation of outcomes, and outcome prediction. The scale demonstrated an internal consistency of ω = 0.79 in this study.

### 2.4. Statistical Analyses

Descriptive statistics, including means, standard deviations, frequencies, and percentages, were calculated to analyze participants’ demographic characteristics, gambling behaviors, and gambling-related psychological variables. To assess differences in categorical variables, the chi-square test (χ^2^) was used. Effect sizes were estimated using phi (φ) and Cramer’s *V*, interpreting values as follows: 0.10 as small, 0.30 as moderate, and 0.50 as large effect sizes, or ≥0.06, ≥0.17, and ≥0.29, when degrees of freedom were ≥3 (Cohen, 1988).

For continuous variables, between-group differences were analyzed using the non-parametric Mann–Whitney U test due to non-normal distributions of the data (Kolmogorov–Smirnov test, *p* < 0.05). Effect sizes were calculated using Rosenthal’s *r*, with thresholds of 0.10 (small), 0.30 (moderate), and 0.50 (large).

To examine whether gambling self-efficacy moderated the association between psychological risk factors and gambling severity, moderation analyses were conducted using PROCESS macro version 5.0 (Model 1; Hayes, 2022). Two separate moderation models were estimated. In Model 1, impulsivity was specified as the focal predictor (X), gambling self-efficacy as the moderator, and problem gambling severity (PGSI total score) as the outcome, with cognitive distortions included as a covariate. In Model 2, cognitive distortions were specified as the focal predictor, gambling self-efficacy as the moderator, and PGSI as the outcome, with impulsivity included as a covariate. In both models, the number of gambling activities, maximum gambling frequency, and education level were entered as additional covariates. All continuous predictors were mean-centered prior to analysis. Estimates were computed using heteroskedasticity-consistent standard errors (HC3). All analyses were performed using SPSS v.26, and significance was set at *p* < 0.05.

## 3. Results

### 3.1. Sociodemographic Profile of Individuals with Low and High Gambling Self-Efficacy

Based on the BSCQ-G scores, 51.5% (*n* = 474) of the 921 participants were classified as having low gambling self-efficacy (LSE, BSCQ-G scores < 80%), while 48.5% (*n* = 447) were classified as having high gambling self-efficacy (HSE, BSCQ-G scores ≥ 80%). The sociodemographic characteristics of both groups are presented in Table 1.

No statistically significant differences were observed between the low and high self-efficacy groups in sex, age, employment status, or income. Educational level was the only sociodemographic characteristic that differed between groups (*p* = 0.032), with a higher proportion of participants with higher education in the HSE group (55.0%; *n* = 246) compared with the LSE group (46.4%, *n* = 220), although the effect size was small (Cramer’s *V* = 0.10).

### 3.2. Differences Between Low and High Gambling Self-Efficacy Groups in Gambling Involvement

As shown in Table 2, the LSE group engaged in a significantly higher number of gambling activities over the past year compared to the HSE group (*p* < 0.001), with a small effect size (*r* = 0.13). Gambling frequency also differed significantly between groups (*p* < 0.001), with a moderate effect size (Cramer’s *V* = 0.17). Gambling less than once a month was more common in the HSE group (49.2%, *n* = 220), compared to the LSE group (36.5%, *n* = 173), whereas weekly gambling was more prevalent among individuals with LSE (26.2%, *n* = 124) than among those with HSE (14.1%, *n* = 63). In addition to differences in traditional gambling involvement, engagement in gambling-like activities, such as betting within video games and on streaming platforms, was also more frequent among participants with LSE (10.5%, *n* = 50) than among those with HSE (5.4%, *n* = 24; *p* = 0.006). Participants in the LSE group further reported involvement in a greater number of these activities (*p* = 0.004). Both differences were statistically significant, but of small magnitude (φ = 0.10 and *r* = 0.10, respectively). No significant differences (*p* > 0.05) were observed between groups in the preferred form of gambling (land-based vs. online).

Regarding problem gambling indicators, a significantly higher proportion of participants with LSE met the criteria for problem gambling (PGSI ≥ 5; 12.2%, *n* = 58), compared to those with HSE (0.9%, *n* = 4; *p* < 0.001), with a small effect size (φ = 0.23). Consistently, participants with LSE scored significantly higher on overall problem gambling severity (PGSI total score) than those with high self-efficacy (*M_LSE_* = 1.8, *SD_LSE_* = 3.8 vs. *M_HSE_* = 0.4, *SD_HSE_* = 1.8; *p* < 0.001), again with a small effect size (*r* = 0.25).

### 3.3. Differences Between Low and High Gambling Self-Efficacy Groups in Gambling-Related Psychological Characteristics

Participants with LSE exhibited significantly higher overall impulsivity scores (*M_LSE_* = 44.0; *SD_LSE_* = 8.3) than those reporting HSE (*M_HSE_* = 38.2, *SD_HSE_* = 7.7; *p* < 0.001), with a moderate effect size (*r* = 0.35). This pattern was consistent across all impulsivity dimensions, with the largest effects observed for positive urgency (*r* = 0.31), followed by sensation seeking (*r* = 0.23) and negative urgency (*r* = 0.22), and smaller effects for lack of premeditation and lack of perseverance (both *r* = 0.20).

Differences were also observed in gambling-related cognitive distortions. Individuals with LSE reported significantly higher levels of cognitive distortions than those with HSE (*M_LSE_* =16.8, *SD_LSE_* = 4.3 vs. *M_HSE_* = 14.7, *SD_HSE_* = 3.9; *p* < 0.001), with a small effect size (*r* = 0.27). When examining specific types of cognitive distortions, the largest differences were observed for illusory correlation (*r* = 0.25), followed by illusion of control and biased evaluation of outcomes (both *r* = 0.21), and outcome prediction (*r* = 0.14). In contrast, no significant differences were found between groups for self-correcting randomness or beliefs about luck as responsible for gambling outcomes (*p* > 0.05).

### 3.4. Moderating Effects of Gambling Self-Efficacy on the Relationship Between Psychological Risk Factors and Gambling Severity

As shown in Table 3, the model including impulsivity as a focal predictor accounted for 42.2% of the variance in gambling severity (R^2^ = 0.427, Adjusted R^2^ = 0.422, *p* < 0.001). Impulsivity was positively associated with PGSI scores (b = 0.045, 95% CI [0.026, 0.063], *p* < 0.001). Gambling self-efficacy was not independently associated with PGSI (b = −0.003, 95% CI [−0.007, 0.001], *p* = 0.095). The interaction between impulsivity and gambling self-efficacy was statistically significant (b = −0.0006, 95% CI [−0.001, −0.0001], *p* = 0.030; ΔR^2^ = 0.0024), indicating that the association between impulsivity and gambling severity varied as a function of self-efficacy.

**Figure 1:**
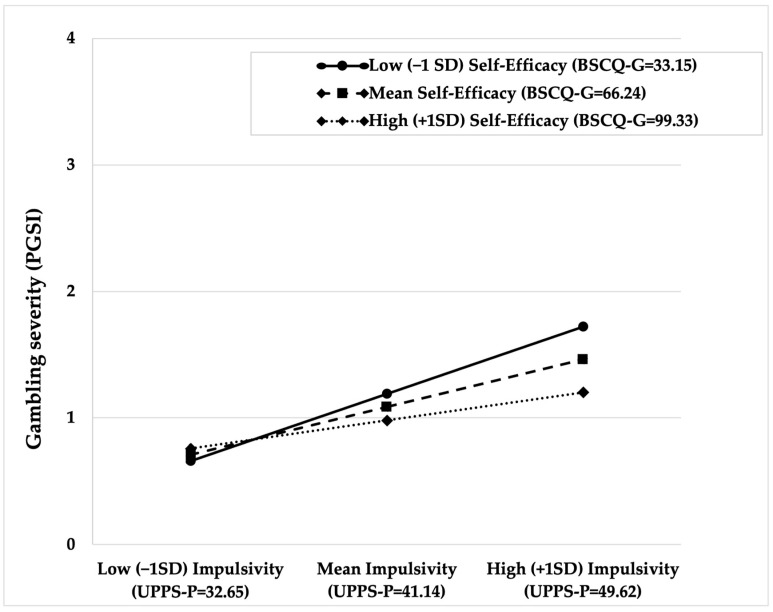
Predicted PGSI scores across impulsivity levels at low (−1 SD), mean, and high (+1 SD) gambling self-efficacy. Covariates (cognitive distortions, gambling involvement indicators, and education level) were held constant at their sample means. The y-axis was restricted to 0–4, rather than the full theoretical PGSI scale (0–27), to facilitate visual interpretation of the conditional effects.

Simple slope analyses showed that the association between impulsivity and PGSI was the strongest at low levels of gambling self-efficacy (−1 SD; b = 0.063, SE = 0.014, 95% CI [0.034, 0.091], *p* < 0.001), and progressively attenuated at the mean (b = 0.045, SE = 0.009, 95% CI [0.026, 0.063], *p* < 0.001), and became weaker at high levels of self-efficacy (+1 SD; b = 0.026, SE = 0.010, 95% CI [0.006, 0.046], *p* = 0.010) (see Figure 1). No Johnson–Neyman transition points were identified within the observed range of the moderator.

In the model including cognitive distortions as the focal predictor, 42.3% of the variance in PGSI was explained (R^2^ = 0.428, Adjusted R^2^ = 0.423, *p* < 0.001). Cognitive distortions were positively associated with gambling severity (b = 0.120, 95% CI [0.070, 0.170], *p* < 0.001), while gambling self-efficacy was not independently associated with PGSI (b = −0.003, 95% CI [−0.007, 0.001], *p* = 0.115). The interaction between cognitive distortions and self-efficacy did not reach statistical significance (b = −0.0013, 95% CI [−0.0027, 0.0001], *p* = 0.062; ΔR^2^ = 0.0031).

Across both models, gambling involvement indicators (i.e., past-year frequency of gambling involvement, number of gambling activities participated in, and number of gambling activities within video games participated in) were consistently associated with higher PGSI scores (*p* < 0.05), whereas education level was not significantly related to gambling severity.

## 4. Discussion

Given the critical role of self-efficacy in the context of addictive behaviors, this study examined differences between individuals with low and high gambling self-efficacy in a general population sample, focusing on gambling involvement, participation in gambling-like activities, sociodemographic characteristics, impulsivity, and cognitive distortions.

Individuals with low gambling self-efficacy showed a broader and more intensive pattern of gambling involvement, participating in a greater number of gambling activities and more frequently than their high self-efficacy counterparts. Notably, this group also showed higher engagement in gambling-like activities within video games and streaming platforms. This finding is consistent with previous studies indicating that low self-efficacy is linked to a greater and more diversified involvement in gambling practices (Hawker et al., 2021; He & Tong, 2024; Quinn et al., 2019), suggesting that reduced confidence in resisting gambling urges across different risk situations may also be associated with engagement in activities beyond traditional gambling formats. These results also align with previous research indicating that lower self-efficacy is associated with higher gambling frequency (León-Jariego et al., 2020; Quinn et al., 2019), whereas individuals with greater self-efficacy in resisting gambling opportunities tend to gamble less frequently (León-Jariego et al., 2020).

Consistent with these behavioral patterns, problem gambling was substantially more prevalent among individuals with low gambling self-efficacy. These results extend the existing evidence by demonstrating that the association between self-efficacy and problem gambling severity is also observable in the general population, not only among clinical or high-risk groups (Casey et al., 2008; Hawker et al., 2021; He & Tong, 2024; Kaur et al., 2006; Quinn et al., 2019).

In terms of sociodemographic profiles, educational attainment emerged as a distinguishing factor. Individuals with high self-efficacy, in addition to reporting lower gambling involvement, more frequently held university degrees. These findings are consistent with previous research indicating that low self-efficacy is associated with greater involvement in problem gambling (He & Tong, 2024) and that lower educational attainment is both a risk factor for problem gambling (Hing et al., 2022b) and a predictor of gambling-related harm (Browne et al., 2019).

Finally, differences between self-efficacy groups were also evident in psychological characteristics linked to gambling risk. Participants with low gambling self-efficacy exhibited significantly higher levels of overall impulsivity. This association is supported by prior studies linking lower self-efficacy with higher impulsivity (Barbaranelli et al., 2017; Lai et al., 2015; Oei & Goh, 2015; C. S.-K. Tang et al., 2011). Moreover, individuals with low gambling self-efficacy also displayed significantly higher scores in gambling-related cognitive distortions. These results align with research indicating that a stronger illusion of control is associated with greater gambling expectancy bias and lower self-efficacy to resist gambling, both of which are linked to problem gambling (Choi & Kim, 2021; Clark & Wohl, 2022; He & Tong, 2024; Iliceto et al., 2021; Ridder & Deighton, 2022; Stark, 2014; C. S. Tang & Wu, 2012).

In addition to describing differences between low and high self-efficacy profiles, we examined self-efficacy as a continuous moderator between key psychological risk factors and gambling severity (PGSI). Results indicated that gambling self-efficacy attenuated the association between impulsivity and gambling severity, with the relationship being strongest at low self-efficacy levels and progressively weaker as gambling self-efficacy increased. Although the incremental variance explained by the interaction was very small (ΔR^2^ = 0.0024), the pattern of simple slopes suggests a modest conditional association whereby greater perceived capacity to resist gambling urges may attenuate the extent to which impulsivity is associated with gambling severity. This finding is consistent with previous research showing that self-efficacy weakens the impact of psychological risk processes on gambling behavior and related outcomes (Challet-Bouju et al., 2025; Dowling et al., 2021c; Free et al., 2024; Hawker et al., 2021; He & Tong, 2024). Importantly, this conditional effect emerged while accounting for cognitive distortions and multiple indicators of gambling involvement, suggesting that this conditional pattern can be observed even after accounting for differences in overall gambling involvement.

Notably, gambling self-efficacy was not independently associated with gambling severity once impulsivity, cognitive distortions, and involvement indicators were considered simultaneously. This result suggests that, after accounting for these psychological and involvement-related factors, gambling self-efficacy may not operate as an independent protective factor in the prediction of PGSI. However, it may still play a modest conditional role, as it moderated the strength of the association between impulsivity and gambling severity. This pattern is compatible with relapse-prevention and self-regulation models, which conceptualize self-efficacy as a mechanism embedded within risk processes that interacts with individual vulnerabilities and situational demands (Marlatt & Gordon, 1985; Witkiewitz & Marlatt, 2004).

In contrast, gambling self-efficacy did not significantly moderate the association between gambling-related cognitive distortions and gambling severity. Thus, although cognitive distortions were directly associated with gambling severity, the present data do not provide clear evidence that this association varied as a function of perceived urge-resistance capacity. One possible explanation is the operational focus of the constructs assessed. The BSCQ-G measures confidence in resisting gambling urges across high-risk situations, whereas the cognitive distortions scale assesses distorted beliefs about gambling outcomes, such as control, prediction, luck, and randomness. Accordingly, gambling self-efficacy as measured in this study may be more closely aligned with urge-resistance and behavioral regulation than with variation in the association between distorted gambling beliefs and severity. From this perspective, strengthening gambling self-efficacy could be particularly relevant for individuals whose gambling severity is linked to impulsivity-related vulnerabilities, whereas gambling-related cognitive distortions may require more direct cognitive approaches, such as identifying and restructuring erroneous beliefs about gambling outcomes (Donati et al., 2018). This interpretation should remain cautious, as the two interaction effects were not formally compared and the cognitive distortions interaction approached, but did not reach, conventional statistical significance.

Overall, these findings extend previous work on gambling self-efficacy, which has largely focused on clinical or treatment-seeking groups, highlighting the importance of considering gambling self-efficacy also in the early translation of vulnerability into harm among non-clinical samples, rather than solely within recovery or relapse processes. Finally, it is important to note that the situational gambling self-efficacy measure used in this study (BSCQ-G; Coloma-Carmona et al., 2026a) has shown meaningful associations with gambling severity and involvement indicators in general-population validation work. In that validation study, the BSCQ-G cutoff (BSCQ-G < 80%) showed high sensitivity for identifying individuals with gambling problems (PGSI ≥ 5), although its moderate specificity indicated that high self-efficacy does not fully rule out gambling-related problems. In the present study, the absence of a direct association between gambling self-efficacy and PGSI in the multivariate models of the present study does not contradict this prior evidence. Instead, the examination of self-efficacy as a moderator helps to clarify the potential processes underlying these associations and suggests that lower perceived resistance capacity may signal conditions in which dispositional vulnerabilities, particularly impulsivity, are more likely to translate into gambling severity. In this regard, the present findings extend prior validation work by moving from identifying susceptibility to clarifying how that susceptibility is expressed within broader vulnerability pathways.

Nonetheless, some study limitations should be considered when interpreting these findings. First, the comparative cross-sectional design identifies associations between variables but does not allow for causal or temporal inferences. Longitudinal studies are therefore needed to clarify the temporal relationships between gambling self-efficacy, psychological characteristics, and the development of gambling-related problems. Second, although the sample included adults who gamble from the Spanish general population, the findings may not be generalizable beyond this context. Reliance on an online panel for data collection may have led to a disproportionate representation of online gambling behavior. Nevertheless, online panels have been recognized as effective tools for investigating gambling patterns in digital environments (Allami et al., 2024; Hawker et al., 2025; Wardle & Tipping, 2023). Third, the sample covered a broad adult age range, from emerging adulthood to later adulthood. Because impulsivity-related processes may vary across developmental stages, future studies should examine whether the observed associations differ across age groups or are moderated by age. Finally, data were collected over a one-month period between March and April 2022. However, gambling involvement was assessed using a past-year reference period that encompassed all months, thereby minimizing the possibility that the findings are influenced by short-term seasonal fluctuations. Nevertheless, unmeasured contextual influences related to the data-collection period cannot be completely ruled out.

## 5. Conclusions

Individuals with low gambling self-efficacy exhibited broader and more intensive patterns of gambling involvement, including participation in a greater number of gambling activities and more frequent engagement in gambling-like activities embedded in video games and streaming platforms. They also showed higher levels of problem gambling severity, impulsivity, and gambling-related cognitive distortions compared to individuals with high gambling self-efficacy.

Together, these findings underscore the relevance of gambling self-efficacy as a key factor associated with both behavioral and psychological dimensions of gambling-related harm in the general population, while also indicating that its role extends beyond simple association, shaping how underlying vulnerabilities, in particular impulsivity, translate into gambling severity.

## Figures and Tables

**Table 1:** Socio-demographic profile of individuals with low and high gambling self-efficacy.

	Total (*N* = 921)	Low Self-Efficacy BSCQ-G < 80% (*n* = 474)	High Self-Efficacy BSCQ-G ≥ 80% (*n* = 447)	Statistic (*p* Value)	^†^ ES
Sex, % (*n*)				χ^2^ = 0.427 (0.514)	
Female	47.8 (440)	46.6 (221)	49.0 (219)		
Male	52.2 (481)	53.4 (253)	51.0 (228)		0.02
Age (years), mean (SD)	40.0 (12.1)	39.3(12.1)	40.8 (12.1)	Z = −1.827 (0.068)	0.06
Education level, % (*n*)					
None	0.3 (3)	0.4 (2) ^a^	0.2 (1) ^a^	χ^2^ = 8.818 (0.032) *	0.10
Elementary/primary	5 (46)	6.3 (30) ^a^	3.6 (16) ^a^		
Secondary/technical	44.1 (406)	46.8 (222) ^a^	41.2 (184) ^a^		
Higher	50.6 (466)	46.4 (220) ^a^	55.0 (246) ^b^		
Employment status, % (*n*)					
Student	9.3 (86)	9.9 (47)	8.7 (39)	χ^2^ = 1.396 (0.706)	0.04
Employed	69.3 (638)	69.6 (330)	68.9 (308)		
Unemployed/domestic work	16.6 (153)	15.4 (73)	17.9 (80)		
Retired	4.8 (44)	5.1 (24)	4.5 (20)		
Income (€), % (*n*) ^††^					
<1000	20.9 (146)	21.8 (79)	19.8 (67)	χ^2^ = 1.389 (0.499)	0.05
1000–1999	55.9 (391)	56.6 (205)	55.0 (186)		
≥2000	23.3 (163)	21.5 (78)	25.1 (85)		

SD: standard deviation; %: percentage; *n*: number of cases; ES: effect size. Within each row, different superscript letters indicate statistically significant differences between groups. ^†^ Effect sizes, Cramer’s *V*/φ and Rosenthal’s *r*, were calculated for the non-continuous and continuous variables, respectively. ^††^ Missing data: Do not know/no response (*n* = 221). * *p* < 0.05.

**Table 2:** Gambling behaviors and gambling-related psychological characteristics of individuals with low and high gambling self-efficacy.

	Total (*N* = 921)	Low Self-Efficacy BSCQ-G < 80% (*n* = 474)	High Self-Efficacy BSCQ-G ≥ 80% (*n* = 447)	Statistic (*p* Value)	^†^ ES
No. of gambling activities, mean (SD)	1.5 (1.1)	1.6 (1.2)	1.4 (1.0)	Z = −3.860 (<0.001) **	0.13
Past-year frequency of gambling involvement, % (*n*) ^††^					
Less than once a month	42.7 (393)	36.5 (173) ^a^	49.2 (220) ^b^	χ^2^ = 25.247 (<0.001) **	0.17
Monthly	34.7 (320)	35 (166) ^a^	34.5 (154) ^a^		
Weekly	20.3 (187)	26.2 (124) ^a^	14.1 (63) ^b^		
Daily	2.3 (21)	2.3 (11) ^a^	2.2 (10) ^a^		
Preferred form of gambling, % (*n*) ^††^					
Land-based	64.8 (597)	64.3 (305)	68.7 (307)	χ^2^ = 1.749 (0.186)	0.05
Online	35.2 (324)	35.7 (169)	31.3 (140)		
Participation in gambling-like activities					
Gambling within video games/streaming platforms, % (*n*)	8 (74)	10.5 (50)	5.4 (24)	χ^2^ = 7.666 (0.006) **	0.10
No. of gambling activities within video games/streaming platforms, mean (SD)	0.2 (0.6)	0.2 (0.7)	0.1 (0.5)	Z = −2.907 (0.004) **	0.10
Problem gambling					
Severity (PGSI total score), mean (SD)	1.1 (3.1)	1.8 (3.8)	0.4 (1.8)	Z = −7.502 (<0.001) **	0.25
PGSI score ≥ 5, % (*n*)	6.7 (62)	12.2 (58)	0.9 (4)	χ^2^ = 45.341 (<0.001) **	0.23
Impulsivity (UPPS-P), mean (SD)					
Total score	41.1 (8.5)	44.0 (8.3)	38.2 (7.7)	Z = −10.478 (<0.001) **	0.35
Negative urgency	9.4 (2.8)	9.9 (2.8)	8.8 (2.7)	Z = −6.649 (<0.001) **	0.22
Lack of premeditation	6.8 (2.0)	7.2 (2.1)	6.4 (1.8)	Z = −6.137 (<0.001) **	0.20
Lack of perseverance	7.1 (2.2)	7.5 (2.3)	6.6 (2.1)	Z = −6.104 (<0.001) **	0.20
Sensation seeking	8.9 (2.7)	9.5 (2.7)	8.3 (2.6)	Z = −7.077 (<0.001) **	0.23
Positive urgency	9.0 (2.5)	9.8 (2.4)	8.3 (2.3)	Z = −9.330 (<0.001) **	0.31
Gambling-related cognitive distortions, mean (SD)					
Total score	15.8 (4.2)	16.8 (4.3)	14.7 (3.9)	Z = −8.134 (<0.001) **	0.27
Illusion of control	2.9 (1.1)	3.1 (1.2)	2.7 (1.0)	Z = −6.446 (<0.001) **	0.21
Biased evaluation of outcomes	1.6 (0.7)	1.7 (0.8)	1.4 (0.7)	Z = −6.340 (<0.001) **	0.21
Illusory correlation	3.1 (1.3)	3.4 (1.4)	2.8 (1.1)	Z = −7.720 (<0.001) **	0.25
Self-correcting randomness	2.2 (1.0)	2.2 (1.0)	2.2(1.0)	Z = −0.487 (0.627)	0.02
Outcome prediction	1.7 (0.8)	1.8 (0.8)	1.6 (0.8)	Z = −4.241 (<0.001) **	0.14
Luck as responsible for outcomes	3.8 (1.2)	3.8 (1.3)	3.8 (1.2)	Z = −0.457 (0.648)	0.02

SD: standard deviation; %: percentage; *n*: number of cases; ES: effect size. Within each row, different superscript letters indicate statistically significant differences between groups. ^†^ Effect sizes, Cramer’s *V*/φ and Rosenthal’s *r*, were obtained for the non-continuous and continuous variables, respectively. ** *p* < 0.01. ^††^ The frequency of gambling involvement refers to the highest frequency for any gambling activity. The preferred form was defined by the setting (online vs. land-based), comprising the majority of reported activities.

**Table 3:** Moderation models predicting gambling severity (PGSI total score) with gambling self-efficacy as a moderator.

Predictor	Model 1 (Impulsivity as Focal Predictor)			Model 2 (Cognitive Distortions as Focal Predictor)		
	b (SE)	*p* Value	95% CI	b (SE)	*p* Value	95% CI
**Constant**	**−2.052 (0.635)**	**0.001** **	**[−3.299, −0.805]**	**−1.988 (0.647)**	**0.002** **	**[−3.259, −0.718]**
**Focal predictor (X)**						
Impulsivity (total score)	**0.045 (0.009)**	**<0.001** **	**[0.026, 0.063]**	—	—	—
Cognitive distortions (total score)	—	—	—	**0.120 (0.026)**	**<0.001** **	**[0.070, 0.170]**
**Moderator (W)**						
Gambling self-efficacy (total score)	−0.003 (0.002)	0.095	[−0.007, 0.001]	−0.003 (0.002)	0.115	[−0.007, 0.001]
**Interaction (X × W)**	**−0.0006 (0.0003)**	**0.030** *	**[−0.001, −0.0001]**	−0.0013 (0.0007)	0.062	[−0.0027, 0.0001]
**Covariates**						
Cognitive distortions (total score)	**0.123 (0.026)**	**<0.001** **	**[0.072, 0.174]**	—	—	—
Impulsivity (total score)	—	—	—	**0.046 (0.010)**	**<0.001** **	**[0.027, 0.065]**
Past-year frequency of gambling involvement	**0.234 (0.094)**	**0.013** *	**[0.049, 0.417]**	**0.236 (0.094)**	**0.012** *	**[0.052, 0.420]**
No. of gambling activities	**0.532 (0.145)**	**<0.001** **	**[0.247, 0.816]**	**0.547 (0.145)**	**<0.001** **	**[0.264, 0.831]**
No. of gambling activities within video games	**1.915 (0.377)**	**<0.001** **	**[1.174, 2.655]**	**1.914 (0.377)**	**<0.001** **	**[1.174, 2.654]**
Education level	−0.023 (0.147)	0.874	[−0.313, 0.266]	−0.031 (0.147)	0.835	[−0.319, 0.258]
**Model fit**						
R^2^ (Adjusted R^2^)	0.427 (0.422)			0.428 (0.423)		
F (8, 912)	**21.024**	**<0.001** **		**20.721**	**<0.001** **	

Note. *N* = 921. b = unstandardized coefficient. SE = heteroskedasticity-consistent standard error (HC3). CI = 95% confidence interval. In Model 1, impulsivity was specified as the focal predictor, and cognitive distortions were included as a covariate. In Model 2, cognitive distortions were specified as the focal predictor, and impulsivity was included as a covariate. Both models included gambling severity (PGSI total score) as the outcome variable and additionally controlled for gambling involvement indicators and education level. Significant effects (*p* < 0.05) are shown in bold. * *p* < 0.05, ** *p* < 0.01.

## Data Availability

The data are not publicly available due to ethical reasons.
